# Bioactivation of food genotoxicants 5-hydroxymethylfurfural and furfuryl alcohol by sulfotransferases from human, mouse and rat: a comparative study

**DOI:** 10.1007/s00204-014-1392-6

**Published:** 2014-11-05

**Authors:** Benjamin Sachse, Walter Meinl, Yasmin Sommer, Hansruedi Glatt, Albrecht Seidel, Bernhard H. Monien

**Affiliations:** 1 Research Group Genotoxic Food Contaminants, German Institute of Human Nutrition (DIfE) Potsdam-Rehbrücke, Arthur-Scheunert-Allee 114-116, 14558 Nuthetal, Germany; 2 Department of Nutritional Toxicology, German Institute of Human Nutrition (DIfE) Potsdam-Rehbrücke, 14558 Nuthetal, Germany; 3 Biochemical Institute for Environmental Carcinogens, Prof. Dr. Gernot Grimmer-Foundation, 22927 Grosshansdorf, Germany; 4 Present Address: German Federal Institute for Risk Assessment (BfR), Max-Dohrn-Strasse 8-10, 10589 Berlin, Germany

**Keywords:** Food carcinogens, 5-Hydroxymethylfurfural, Furfuryl alcohol, Sulfotransferase, UPLC-MS/MS

## Abstract

5-Hydroxymethylfurfural (HMF) and furfuryl alcohol (FFA) are moderately potent rodent carcinogens that are present in thermally processed foodstuffs. The carcinogenic effects were hypothesized to originate from sulfotransferase (SULT)-mediated bioactivation yielding DNA-reactive and mutagenic sulfate esters, a confirmed metabolic pathway of HMF and FFA in mice. It is known that orthologous SULT forms substantially differ in substrate specificity and tissue distribution. This could influence HMF- and FFA-induced carcinogenic effects. Here, we studied HMF and FFA sulfoconjugation by 30 individual SULT forms of humans, mice and rats. The catalytic efficiencies (*k*_cat_/*K*_M_) of HMF sulfoconjugation of human SULT1A1 (13.7 s^−1^ M^−1^), mouse Sult1a1 (15.8 s^−1^ M^−1^) and 1d1 (4.8 s^−1^ M^−1^) and rat Sult1a1 (5.3 s^−1^ M^−1^) were considerably higher than those of all other SULT forms investigated (≤0.73 s^−1 ^M^−1^). FFA sulfoconjugation was monitored using adenosine as a nucleophilic scavenger for the reactive 2-sulfoxymethylfuran (*t*_1/2_ = 20 s at 37 °C). The resulting adduct *N* ^6^-((furan-2-yl)methyl)-adenosine (*N* ^6^-MF-A) was quantified by isotope-dilution UPLC-MS/MS. The rates of *N* ^6^-MF-A formation showed that hSULT1A1 and its orthologues in mice and rats were also the most important contributors to FFA sulfoconjugation in each of the species. Taken together, the catalytic capacity of hSULT1A1 is comparable to that of mSult1a1 in mice, the species in which carcinogenic effects of HMF and FFA were detected. This is of primary concern due to the expression of hSULT1A1 in many different tissues.

**Electronic supplementary material:**

The online version of this article (doi:10.1007/s00204-014-1392-6) contains supplementary material, which is available to authorized users.

## Introduction

5-Hydroxymethylfurfural (HMF) and furfuryl alcohol (FFA) are furan derivatives present in many foodstuffs. HMF is formed from reducing sugars in the Maillard reaction and by acid-catalyzed dehydration. It occurs in carbohydrate-rich foods (Husoy et al. 2008; Murkovic and Swasti 2013), coffee, fruit juices and other beverage products (Murkovic and Pichler 2006; Teixido et al. 2006) and cigarette smoke (Crump and Gardner 1989). Estimates of the mean daily dietary intake are in the range of 4–30 mg HMF per person (Federal Institute of Risk Assessment (BfR) 2011). The structural congener FFA originates from cyclization and aromatization of 2-deoxypentose, a key intermediate of heat-induced degradation of glucose or fructose (Brands and van Boekel 2001). It is found in thermally processed foods such as cocoa, tea, coffee, dehydrated orange products, cooked meat and milk products (Maga 1979; Murkovic and Swasti 2013). High concentrations of FFA in coffee (267–1,490 µg/g in three different brands) result in intake amounts of several mg FFA per serving (Murkovic and Swasti 2013). Humans are also exposed by inhalation, particularly in occupational environments due to FFA use in the manufacturing of furan resins (Ahman et al. 1991), but also in residencies, where high levels of FFA (0.4–500 µg/g dust) were found in particulate matter (Nilsson et al. 2005).

HMF and FFA are rodent carcinogens. It was reported that HMF caused the formation of aberrant crypt foci in the colon of rats (Zhang et al. 1993) and neonatal *Min*/+ mice (Svendsen et al. 2009) and initiated papillomas in mouse skin (Surh et al. 1994). Oral administration of 188 mg HMF/kg for 104 weeks led to increased incidences of hepatocellular adenomas in female B6C3F1 mice compared with the control animals (National Toxicology Program 2008). The exposure of male B6C3F1 mice to 32 ppm FFA in the air (equivalent to 60 mg FFA/kg bw/day) induced renal tubule neoplasms, which were also observed in some of the female rats (National Toxicology Program 1999). The mutagenic potential of HMF (Glatt et al. 2005; Janzowski et al. 2000; National Toxicology Program 2008) and FFA (Aeschbacher et al. 1989; National Toxicology Program 1999) was small in standard in vitro tests. However, the mutagenic and genotoxic activity observed in sulfotransferase (SULT)-proficient bacteria (Glatt et al. 2011) and mammalian cell lines suggests that the tumor-initiating potential of HMF and FFA may depend, in part or completely, on sulfoconjugation. This hypothesis was lend further credibility by a toxicokinetic study demonstrating that HMF is converted into mutagenic 5-sulfoxymethylfurfural (SMF) in FVB/N mice (Monien et al. 2009) and that 2-methylfuran adducts such as *N* ^2^-((furan-2-yl)methyl)-2′-deoxyguanosine were detected in DNA from liver, kidney and lung of FVB/N mice that received FFA in the drinking water over 4 weeks (Monien et al. 2011).

It is disturbing that the margins between the carcinogenic doses found in animal experiments and the estimated mean intake values for HMF and FFA are relatively small. The dose of 188 mg HMF/kg body weight significantly increased the incidence of liver adenoma in female mice in a two-year bioassay. This is merely 440- to 3,300-fold higher compared with the human HMF uptake {calculated with 70 kg human body weight and the HMF uptake range of 4–30 mg per person given by the German Federal Institute of Risk Assessment (BfR)] (Federal Institute of Risk Assessment (BfR) 2011; National Toxicology Program 2008). The European Food Safety Authority (EFSA) stated that a margin of exposure below 10,000 indicates a high priority for risk assessment, if the carcinogenicity was based on a genotoxic mechanism (European Food Safety Authority 2005). Moreover, due to considerable differences in SULT expression and substrate specificities of orthologous SULT forms between species (Glatt 2002), human sensitivity to HMF- and FFA-induced effects may differ from that of rodents. Here, we present a comprehensive overview about sulfoconjugation of HMF and FFA by individual SULTs of human and rodent origin using cytosolic preparations from SULT-expressing *Salmonella typhimurium* TA1538 strains.

## Materials and methods

### Chemicals

FFA was purchased from Merck Schuchardt (Hohenbrunn, Germany). HMF, adenosine, ammonium bicarbonate and all other reagents (analytical grade) were from Sigma-Aldrich (Taufkirchen, Germany). HPLC-grade methanol, 2-propanol and formic acid were from Carl Roth GmbH (Karlsruhe, Germany), and HPLC-grade water was prepared using a Milli-Q Integral Water Purification System (Millipore Merck, Darmstadt, Germany). Stable isotope-labeled [^15^N_5_]adenosine was from Silantes GmbH (Munich, Germany). Sulfation reactions of HMF and FFA yielding SMF (Monien et al. 2009) and 2-sulfoxymethylfuran (Monien et al. 2011), respectively, were conducted as described. The cofactor 3′-phosphoadenosine-5′-phosphosulfate (PAPS) was prepared using human PAPS synthetase 2 expressed in *Escherichia coli* and was purified using anion-exchange chromatography (purity > 99.0 %) (Muckel et al. 2001).

### Synthesis of *N*^6^-((furan-2-yl)methyl)-adenosine (*N*^6^-MF-A) and [^15^N_5_]*N*^6^-((furan-2-yl)methyl)-adenosine ([^15^N_5_]*N*^6^-MF-A)

The ribonucleoside adduct of FFA was prepared by dissolving 100 mg adenosine (374 µmol) in 50 ml sodium phosphate buffer (pH 8.0) together with 80 mg (400 µmol) of freshly prepared 2-sulfoxymethylfuran (Monien et al. 2011). The solution was stirred for 15 min at 37 °C. The product mixture was subjected to HPLC purification using a Prep LC 150 (Waters, Eschborn, Germany) coupled to a PDA detector 996 (Waters) and a preparative column SunFire C18 OBD (5 µm, Waters). The product was eluted with 5 % methanol (solvent A) and 75 % methanol (solvent B) using a linear gradient from 100 % solvent A to 10 % solvent A in 20 min at a flow rate of 20 ml/min. The product containing fractions were pooled and freeze-dried. The purity of the product was >99 % as determined by UPLC-UV-MS/MS (MS (ESI^+^) *m/z* = 348.1 [M + H]^+^). ^1^H-NMR of *N* ^6^-MF-A (300 MHz, dimethyl sulfoxide-*d*_6_) *δ* [ppm]: 8,40 (s, 1H, *H8*), 8,26 (bs, 1H, N-*H*), 8,15 (s, 1H, *H2*); 7,55 (s, 1H, *H5″*), 6,37–6,38 (d, 1H, *H1′*), 6,24–6,25 (d, 1H, *H4″*), 5,90–5,91 (d, 1H, *H3″*), 5,49–5,50 (m, 1H, O-*H2′*), 5,42–5,44 (m, 1H, O-*H5′*), 5,24 (m, 1H, O-*H3′*), 4,62–4,63 (m, 2H, N-*CH2*); 4,60–4,61 (m, 1H, *H3′*), 4,15–4,16 (m, 1H, *H2′*), 3,97–3,98 (m, 1H, *H4′*), 3,74–3,75 (m, 1H, *H5′*), 3,67–3,69 (m, 1H, *H5′* ^*2*^). The stable isotope-labeled substance [^15^N_5_]*N* ^6^-MF-A was prepared in the same manner downscaling the reaction by factor 50.

### Cytosolic preparations of SULT-expressing *S. typhimurium* TA1538

Cytosolic fractions containing individual SULT forms were prepared from transgenic *S. typhimurium* TA1538. Table S1 in the Supplementary Material provides a summary of the thirty SULTs used in the current study together with references describing the cloning of the respective *S. typhimurium* TA1538 strains. The expressed SULT forms are denoted with the prefixes h, m and r for human, mouse and rat enzymes, respectively. Bacteria were grown in Luria Broth medium containing ampicillin (100 µg/ml) by stirring for 8 h at 200 revolutions/min at 37 °C. The bacterial suspensions were centrifuged at 5,000 *g* for 10 min. The sediments were washed twice with 25 ml of 10 mM potassium phosphate buffer (pH 7.4)/50 mM potassium chloride (KCP buffer) and centrifuged again. The pellets were taken up in 800 µl KCP buffer and sonicated thrice for 15 s with 30 s pauses in between. The soluble fraction was attained by centrifuging for 1 h at 100,000 *g*. The supernatants (in the following termed cytosolic preparations) were frozen at −80 °C. The protein concentrations were determined according to a bicinchoninic acid (BCA) assay protocol. The SULT concentrations in the cytosolic preparations were estimated after electrophoretic separation and immunodetection using inclusion bodies of different SULT forms as standards (Teubner et al. 2007). The preparation of SULT inclusion bodies and the immunodetection of SULTs is described in the Supplementary Material, which also contains a representative immunoblot of different amounts of rSult1a1standard and a cytosolic preparation of *S. typhimurium* expressing rSult1a1 (Fig. S1).

### HMF sulfoconjugation assay

The reactions were conducted in mixtures of 100 µl containing 25–200 µg protein, 50 mM potassium phosphate buffer (pH 7.4), 5 mM MgCl_2_, 50 µM PAPS and different concentrations of HMF at 37 °C. Preliminary experiments ensured that measurements were recorded under ‘linear’ conditions, i.e., doubling of protein concentration or incubation time was doubling the amount of sulfate ester formed. The reactions were stopped after 30 min by adding 300 µl ice-cold 2-propanol, mixed and centrifuged at 15,000 *g* for 10 min.

SMF concentrations of the clear supernatants were analyzed by UPLC-MS/MS. The protocol was adapted from the quantification of SMF in plasma samples of HMF-treated mice (Monien et al. 2009). In short, the supernatants were analyzed using a UPLC system (Waters) connected to a Quattro Premier XE tandem quadrupole mass spectrometer (Waters) using the negative electrospray ionization mode. Samples of 4 µl were injected into a HSS T3 column (1.8 µm, 2.1 × 100 mm; Waters) and eluted with an isocratic flow of 0.35 ml/min 10 mM ammonium acetate/methanol (95:5). The fragmentation reactions of SMF yielding two principal ions, the sulfate ion radical (*m/z* = 204.9 → 96.0) and the protonated sulfonate ion (*m/z* = 204.9 → 81.0) were monitored. The tune parameters were as follows: temperature of the electrospray source 120 °C; desolvation temperature 475 °C; desolvation gas nitrogen (800 l/h); cone gas nitrogen (100 l/h); collision gas argon (indicated cell pressure ~5 × 10^−3^ mbar). For the fragmentation of SMF, collision energies were 20 and 23 eV for the transitions *m/z* = 204.9 → 81.0 and *m/z* = 204.9 → 96.0, respectively. The dwell time was set to 100 ms, and capillary voltage was set to 0.35 kV. The cone and RF1 lens voltages were 32 and 0.2 V, respectively. SMF was quantified using an external calibration line that was linear in the concentration range of 1.0 nM (4 fmol/injection) to 2,500 (10 pmol/injection) SMF (*r* ^2^ ≥ 0.99). Data acquisition and handling were performed with MassLynx 4.1 software (Waters). The limit of detection (LOD) for this technique of 2.5 nM SMF has been reported (Monien et al. 2009). In the current study, this corresponds to 10 fmol SMF per injection or a reaction rate of 0.5 pmol SMF/mg/min.

### FFA sulfoconjugation assay

The reaction mixtures were prepared as described for HMF. In addition, they contained 10 mM adenosine as a nucleophilic scavenger. Following incubation for 20 min at 37 °C, sulfoconjugation reactions were stopped by adding 100 µl cold methanol containing 175 fmol of [^15^N_5_]*N* ^6^-MF-A as the internal standard, mixed and centrifuged at 15,000 *g* for 10 min. The clear supernatants were subjected to UPLC-MS/MS analyses. Incubations were performed as quadruplicates. At higher concentrations, FFA reacts spontaneously with adenosine at low, but detectable, levels. To correct for the SULT-independent adduct formation, mixtures with SULT-deficient cytosols were carried along simultaneously in duplicate. Controls were subtracted from the experimental samples.

The ribonucleoside adduct *N* ^6^-((furan-2-yl)methyl)-adenosine (*N* ^6^-MF-A) was analyzed by isotope-dilution UPLC-MS/MS with the same system described above using a HSS T3 column (1.8 µm, 2.1 × 100 mm, Waters). Samples of 8 µl were eluted with 10 mM ammonium bicarbonate buffer, pH 8.0 (A) and methanol (B) using an 6-min gradient from 98 % solvent A to 15 % solvent A at 0.35 ml/min flow rate (Yin et al. 2013). The electrospray interface of the mass spectrometer operated in the positive ion mode. The fragmentations of *N* ^6^-MF-A into the aglycone cation [*N* ^6^-((furan-2-yl)methyl)-adenine-H]^+^ (*m/z* = 348.1 → 216.1), the cleavage of the 2-methylfuranyl cation (*m/z* = 348.1 → 81.0) and the release of [*N* ^6^-methyl-adenine-H]^+^ (*m/z* = 348.1 → 148.0) were monitored together with the corresponding fragmentations of the isotope-labeled internal standard [^15^N_5_]*N* ^6^-MF-A for the formation of the aglycone (*m/z* = 353.1 → 221.1), the 2-methylfuran cation (*m/z* = 353.1 → 81.0) and ^15^N_5_-labeled *N* ^6^-methyl-adenine (*m/z* = 353.1 → 153.0). The *N* ^6^-MF-A content of the incubation mixture was calculated from the ratio of peak areas resulting from the total ion current of all three MRM signals of the analyte. For the fragmentation of *N* ^6^-MF-A ([^15^N_5_]*N* ^6^-MF-A), collision energies were 20, 25 and 35 eV for the transitions *m/z* = 348.1 → 216.1 (*m/z* = 353.1 → 221.1), *m/z* = 348.1 → 148.0 (*m/z* = 353.1 → 153.0) and *m/z* = 348.1 → 81.0 (*m/z* = 353.1 → 81.0), respectively. The tune parameters were as follows: temperature of the electrospray source 110 °C; desolvation temperature 450 °C; desolvation gas nitrogen (850 l/h); cone gas nitrogen (50 l/h); collision gas argon (indicated cell pressure ~5 × 10^−3^ mbar). The dwell time was set to 100 ms. The cone gas voltage and capillary voltage were set to 25 V and 0.7 kV, respectively. The RF1 lens voltage was 0.1 V. Data acquisition and handling were performed with MassLynx 4.1 software (Waters).

The trace amounts of *N* ^6^-MF-A also formed in the absence of SULTs increased with increasing concentrations of FFA in the incubation mixture. Due to this background, the LOD values for the quantification of *N* ^6^-MF-A were determined individually for each of the FFA concentrations from the background integral of the total ion current from all three transitions of *N* ^6^-MF-A monitored at the precise retention time in incubations containing all components and SULT-deficient cytosol from *S. typhimurium*. Only a concentration of *N* ^6^-MF-A exceeding the arithmetic mean area of the daily background signal from 10 to 12 blank incubations by more than twofold was considered to be above the LOD. The resulting LOD values for incubations with 0.1, 1 and 10 mM FFA were 0.18, 0.20 and 0.44 nM *N* ^6^-MF-A, respectively, corresponding to reaction rates of 18, 20 and 44 fmol *N* ^6^-MF-A/mg/min.

## Results

### Quantification of SMF by UPLC-MS/MS

The analytical technique used in this study was adapted from the SMF quantification in plasma samples of HMF-treated mice (Monien et al. 2009). This is beset with two potential sources of inaccuracy. First, SMF is a reactive analyte with half-life times of 114, 126 and 108 min in water, urine and plasma, respectively, at 37 °C (Monien et al. 2009). Here, we chose a reaction time of 30 min. Assuming a continuous linear formation of SMF during the incubation and a half-life of 120 min, about 6 % of newly formed SMF should degrade before stopping the reaction by adding cold 2-propanol. Notably, SMF was more stable in solutions containing 75 % 2-propanol at 4 °C without any detectable degradation within four weeks. SMF was analyzed by UPLC-MS/MS in the MRM mode using the specific fragmentation reactions of the sulfate ion radical (*m/z* = 204.9 → 96.0) and the protonated sulfonate ion (*m/z* = 204.9 → 81.0) (Monien et al. 2009). Representative chromatograms are shown in Fig. S2 of the Supplementary Material. The SMF levels resulting from HMF sulfoconjugation were quantified using SMF solutions at various concentrations as external calibration line prepared in water/2-propanol (1:3), which represents the solvent composition in the incubation mixture following instant stopping and precipitating of proteins. The second source of imprecision of mass spectrometric SMF quantification with an external calibration is the possible ion suppression by residues from bacterial cytosol or buffer salts in the samples. This was tested in a separate experiment measuring the peak areas of six series of solutions with five different SMF concentrations, either prepared with water or with the contents of the incubation mixture including 1 mg/ml protein from a cytosolic preparation of native *S. typhimurium* TA1538. The results, summarized in Table S2 of the Supplementary Material, showed that ion suppression did not interfere with SMF detection.

### Sulfoconjugation of HMF by individual human, mouse and rat SULTs

HMF sulfoconjugation was assessed in cytosolic preparations from various strains of *S. typhimurium* TA1538 expressing individual SULT forms of human, mouse and rat. This allowed including 30 SULT forms in this study without time-consuming, multi-step chromatographic procedures for the purification of individual enzymes. The approach offers two additional advantages. First, we found that SULTs in cytosolic preparations are more stable compared with purified enzymes (Teubner et al. 2007), and, second, consumed cofactor 3′-phosphoadenosine-5′-phosphate (PAP) acts as a SULT inhibitor in assays with purified enzymes, but is rapidly decomposed in cytosolic bacterial preparations. Sulfoconjugation of all 30 SULT forms was assessed using 0.1, 1, 10 and 100 mM HMF (Table 1). Among twelve human SULTs included in the study, nine enzymes were found to catalyze HMF conjugation with highest rates observed for SULT1A1, 1A2, 1A3, 1B1 and 1C2. From seven mouse Sult forms catalyzing HMF sulfoconjugation mSult1a1, 1d1 and 1e1 stood out due to the highest turnover rates. Generally, rat Sults showed low activity toward HMF, the highest activity being found for rSult1c1.

**Table 1:** Rates of sulfoconjugation assayed at four HMF concentrations in cytosolic preparations of *S. typhimurium* TA1538 containing different SULT forms from human, mouse and rat

Species	SULT	Rate (pmol/mg/min)			
		0.1 mM HMF	1 mM HMF	10 mM HMF	100 mM HMF
Human	1A1	20.4 ± 2.8	171 ± 18	641 ± 100	699 ± 170
	1A2	<0.5	15.2 ± 4.5	130 ± 53	628 ± 233
	1A3	1.2 ± 0.1	12.1 ± 1.5	117 ± 12	406 ± 39
	1B1	2.0 ± 0.5	22.2 ± 5.2	133 ± 28	272 ± 49
	1C1	<0.5	<0.5	4.7 ± 0.3	11.0 ± 1.4
	1C2	1.1 ± 0.3	14.0 ± 1.8	115 ± 14	285 ± 71
	1C3	<0.5	1.0 ± 0.1	7.2 ± 0.9	3.6 ± 0.2
	1E1	<0.5	1.0 ± 0.2	5.7 ± 0.8	7.7 ± 1.2
	2A1	<0.5	2.4 ± 0.6	31.1 ± 5.4	201 ± 39
	2B1a	<0.5	<0.5	<0.5	<0.5
	2B1b	<0.5	<0.5	<0.5	<0.5
	4A1	<0.5	<0.5	<0.5	<0.5
Mouse	1a1	76.6 ± 14.4	722 ± 93	2,010 ± 210	1,970 ± 70
	1b1	0.9 ± 0.1	8.2 ± 0.1	66.2 ± 6.8	64.4 ± 5.0
	1c2	<0.5	2.0 ± 0.2	16.2 ± 1.1	92.4 ± 6.7
	1d1	124 ± 33	1,160 ± 260	6,750 ± 1,190	22,400 ± 2,400
	1e1	3.7 ± 0.3	35.5 ± 2.6	226 ± 9	534 ± 52
	2a1	<0.5	1.3 ± 0.2	9.0 ± 0.9	13.1 ± 1.7
	2a2	<0.5	4.4 ± 0.8	32.4 ± 3.2	77.5 ± 11.7
	2a3	<0.5	<0.5	<0.5	<0.5
	2b1b	<0.5	<0.5	<0.5	<0.5
	5a1	<0.5	<0.5	<0.5	<0.5
Rat	1a1	<0.5	6.5 ± 2.1	43.6 ± 13.7	36.1 ± 16.8
	1b1	<0.5	3.4 ± 0.2	25.2 ± 0.4	30.8 ± 1.2
	1c1	2.1 ± 1.3	29.1 ± 9.0	120 ± 39	47.5 ± 27.3
	1c2	<0.5	<0.5	2.3 ± 0.2	15.0 ± 1.1
	2a1	<0.5	1.8 ± 0.1	17.2 ± 0.6	58.9 ± 4.6
	2a3	0.6 ± 0.0	6.2 ± 0.2	62.8 ± 4.6	76.5 ± 8.8
	2a4	<0.5	<0.5	<0.5	<0.5
	2b1	<0.5	<0.5	<0.5	<0.5

Values are mean ± SE of three or four measurements. The limit of detection (signal-to-noise ratio = 4) was 2.5 nM SMF or 10 fmol SMF on column corresponding to a rate of 0.5 pmol/mg/min under the assay conditions used (Monien et al. 2009)

**Fig. 1:**
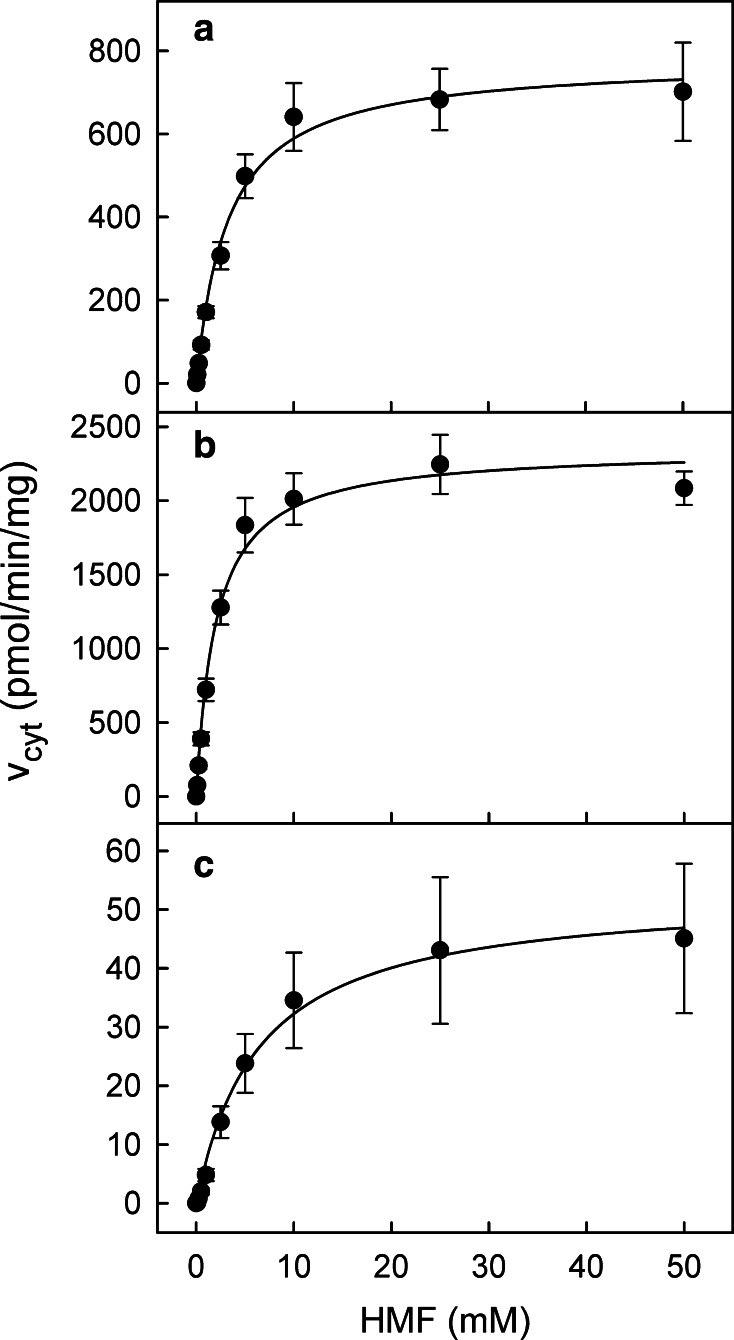
HMF sulfoconjugation by hSULT1A1 (**a**), mSult1a1 (**b**) and rSult1a1 (**c**). The rates at single HMF concentrations are mean ± SE of three or four measurements. Fitting of the data with the Michaelis–Menten model yielded values for the catalytic parameters *K*_M_ and apparent *V*_MAX,cyt_. These were subsequently corrected for the actual concentrations of particular SULT forms in the cytosolic preparations (Table 2)

Eleven of the most active SULTs, five human, three mouse and three rat enzymes, were subjected to a comprehensive kinetic investigation (compare Table 2). The selection was based on the HMF sulfoconjugation rate (Table 1), but also on the quantitative importance of individual SULTs, determined by the tissue distribution in a given adult species and the expression levels reported in relevant organ tissues, especially liver, kidney and colon (Glatt 2002; Riches et al. 2009; Teubner et al. 2002). Current knowledge of these parameters will be discussed. Sulfoconjugation rates of selected enzymes were monitored at ten HMF concentrations. The resulting data were fitted to the Michaelis–Menten equation yielding the Michaelis–Menten constant (*K*_M_) and the maximum rate (*V*_MAX,cyt_). It is of note that *V*_MAX,cyt_ was based on the total protein content in the cytosolic preparations. Figure 1 shows representative kinetics of HMF sulfoconjugation with hSULT1A1, mSult1a1 and rSult1a1 with *K*_M_ values of 3.2 ± 0.4, 2.0 ± 0.3 and 4.9 ± 0.7 mM, respectively. The values of the catalytic parameters *K*_M_ and *V*_MAX,cyt_ of all selected SULT forms are summarized in Table 2. The sulfoconjugation by rSult1c1 was notable due to a decrease in the rate observed at concentrations higher than 50 mM HMF (Supplementary Material Fig. S3). Assuming a substrate inhibitory effect, *K*_M_ and *V*_MAX,cyt_ were derived from the extended Michaelis–Menten equation *V* = *V*_MAX,*cyt*_/(1 + *K*_M_/[HMF] + [HMF]/*K*_I_)that also allowed estimating the dissociation constant (*K*_I_) of the complex formed by rSult1c1-HMF and a second HMF molecule (*K*_I_ = 13.2 ± 3.4 mM).

**Table 2:** Kinetic parameters of HMF sulfoconjugation catalyzed by most prominent SULT forms from human, mouse and rat^a^

Species	SULT	*K*_M_ (mM)^a^	*V*_MAX,cyt_^a,b^ (pmol/mg/min)	SULT concentration (%)	*V*_MAX_^c^ (pmol/mg/min)	*k*_cat_/*K*_M_ (s^−1^ M^−1^)
Human	1A1	3.2 ± 0.4	778 ± 24	1.0	77,800 ± 2,400	13.7
	1A2	56.5 ± 5.4	998 ± 47	1.4	71,300 ± 3,400	0.71
	1A3	16.7 ± 1.8	106 ± 5	1.4^f^	7,570 ± 360	0.26
	1B1	13.3 ± 0.3	310 ± 2	4.2	7,390 ± 40	0.31
	1C2	18.8 ± 5.4	242 ± 24	1.0^f^	24,200 ± 2,400	0.73
Mouse	1a1	2.0 ± 0.3	2,350 ± 80	4.2	55,900 ± 1,800	15.8
	1b1	9.5 ± 1.1	121 ± 5	2.0	6,030 ± 250	0.36
	1d1	31.9 ± 5.7	30,000 ± 2,200	11^d^	273,000 ± 20,000	4.8
Rat	1a1	4.9 ± 0.7	59.5 ± 2.7	0.13	45,800 ± 2,100	5.3
	1b1	15.7 ± 0.9	61.7 ± 1.4	2.5^f^	2,470 ± 60	0.09
	1c1^e^	18.4 ± 4.7	391 ± 76	10^d^	3,910 ± 760	0.12

^a^Values of *K*_M_ and *V*_MAX,cyt_ (±SE) were determined by fitting the HMF sulfoconjugation data averaged from three to five measurements conducted on different occasions with the Michaelis–Menten equation (compare Fig. 1)

^b^Values of *V*_MAX,cyt_ were calculated from whole protein concentrations in the cytosolic preparations

^c^ *V*_MAX_ was calculated from *V*_MAX,cyt_ by correction with the concentrations of individual SULT forms in the cytosolic preparations. These were determined by immunoblotting and comparison with various amounts of inclusion bodies containing >99 % SULT as described by Meinl et al. (2006), unless specified otherwise

^d^The enzyme levels were estimated from the intensity of the SULT band in Coomassie blue-stained polyacrylamide gels after electrophoresis (Meinl et al. 2006)

^e^Due to the inhibition of sulfoconjugation at concentrations ≥ 50 mM HMF, the data were fitted to the equation *V* = *V*_MAX_/(1 + *K*_M_/[HMF] + [HMF]/*K*_I_) yielding *K*_I_ = 13.2 ± 3.4 mM. The fitting curve is shown in the Supplementary Material Figure S3

^f^Meinl et al. (2013)

Fitting of HMF sulfoconjugation rates yielded values of apparent *V*_MAX,cyt_ related to the overall protein content of the cytosolic preparations. In order to correct the *V*_MAX,cyt_, we determined the actual SULT levels of the cytosolic preparations containing the most effective enzymes by immunoblotting using inclusion bodies of homogenous SULTs as reference proteins. Usually, cytosolic SULT concentrations were in the range of those reported previously (Meinl et al. 2006, 2013). The actual reaction rates *V*_MAX_ calculated from *V*_MAX,cyt_ and the SULT content of individual cytosolic preparations are included in Table 2.

The *K*_M_ values of HMF turnover observed for SULT1A1 forms of all three species and rSult1c1 were markedly lower compared with those of all other SULT forms studied. The reaction rates *V*_MAX_ were relatively high with values of 77.8, 55.9 and 45.8 nmol/mg/min for SULT1A1 from human, mouse and rat, respectively. Similar or higher rates were only found for hSULT1A2 (71.3 nmol/mg/min) and mSult1d1 (273 nmol/mg/min). However, the high activities of these enzymes were associated with high *K*_M_ values. The calculation of *k*_cat_/*K*_M_ as a measure for the catalytic efficiency of HMF sulfoconjugation confirmed the predominant role of SULT1A1 in all three species with catalytic efficiencies of 13.7, 15.8 and 5.3 s^−1 ^M^−1^ for human, mouse and rat SULT1A1, respectively.

### Quantification of *N*^6^-((furan-2-yl)methyl)-adenosine (*N*^6^-MF-A) by UPLC-MS/MS

**Fig. 2:**
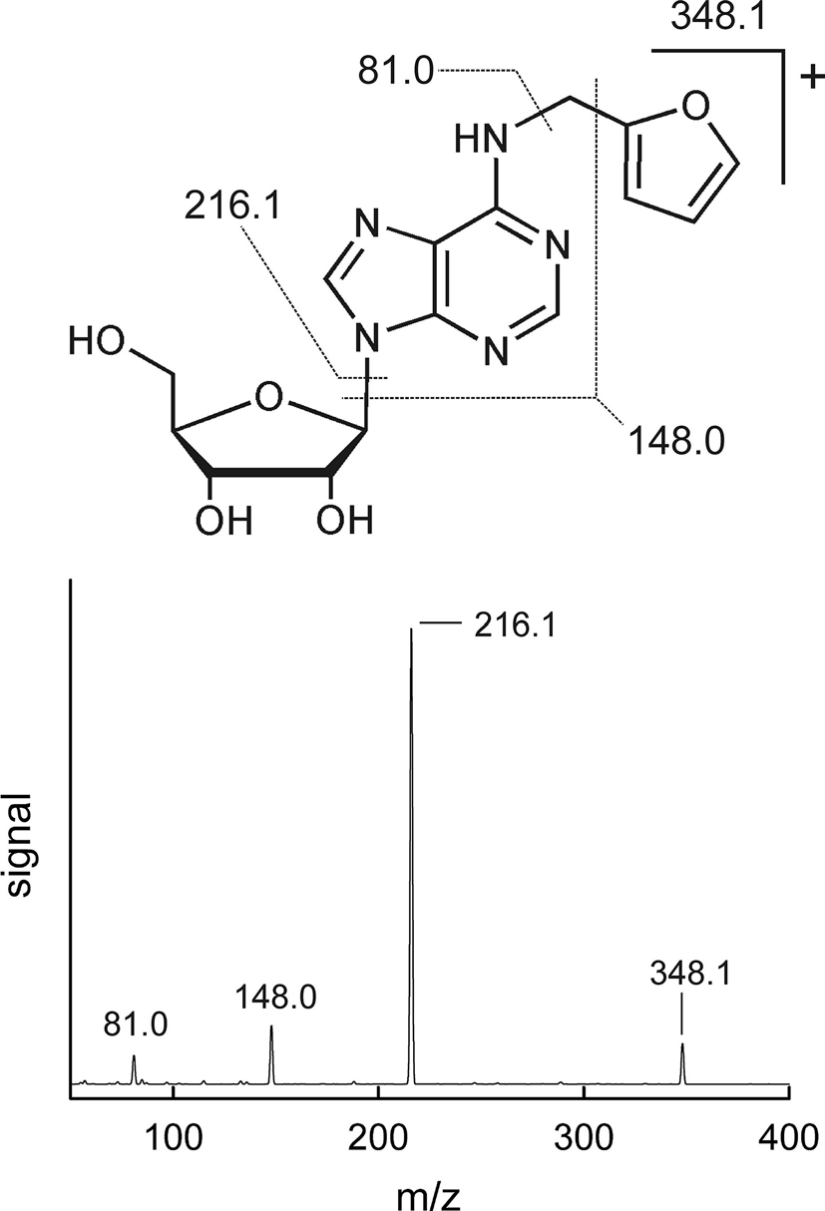
Molecular structure and fragmentation pattern of *N* ^6^-MF-A observed by positive ESI MS/MS collision-induced dissociation. Principal fragmentation ions of *N* ^6^-MF-A were as follows: *m/z* = 216.1 (aglycone of *N* ^6^-MF-A), *m/z* = 81.0 (the cation of methylfuran) and *m/z* = 148.0 (*N* ^6^-methyladenosine)

**Fig. 3:**
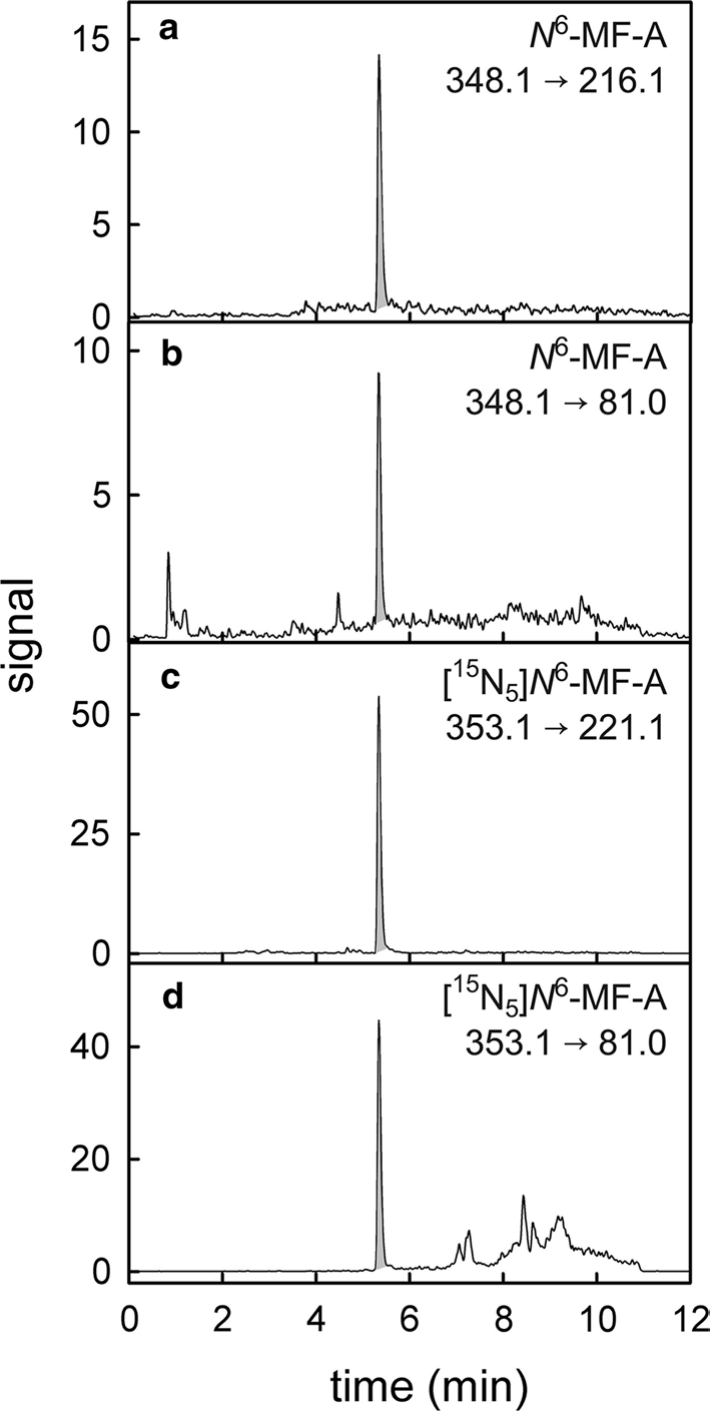
UPLC-MS/MS MRM analysis of *N* ^6^-MF-A in a cytosolic preparation containing hSULT1A1, FFA and adenosine. The chromatograms originate from the fragmentations *m/z* = 348.1 → 216.1 (**a**) and *m/z* = 348.1 → 81.0 (**b**) of *N* ^6^-MF-A and from the transitions *m/z* = 353.1 → 221.1 (**c**) and *m/z* = 353.1 → 81.0 (**d**) of the internal isotope-labeled standard [^15^N_5_]*N* ^6^-MF-A (7.0 fmol/injection)

The protocol of HMF sulfoconjugation by individual SULTs was modified for FFA, because the reactivity of 2-sulfoxymethylfuran (*t*_1/2_ = 20 s in water at 37 °C, (Glatt et al. 2012)) prohibited its direct quantification. We tested various nucleophiles as trapping agents for 2-sulfoxymethylfuran, including adenosine, guanosine, 2′-deoxyadenosine, 2′-deoxyguanosine, Boc-lysine, glutathione and *N*-acetylcysteine. Adenosine was selected because it was well soluble in the incubation medium and formed a single reaction product with 2-sulfoxymethylfuran. The presumed product *N* ^6^-MF-A was synthesized via a biomimetic approach. The mass spectrometric collision-induced dissociation showed the expected fragmentation patterns (Fig. 2): the cleavage of the 2-methylfuran cation (*m/z* = 348.1 → 81.0), the neutral loss of the ribose unit (*m/z* = 348.1 → 216.1) and the release of the positively charged *N* ^6^-methylated adenine (*m/z* = 348.1 → 148.0). The ^1^H-NMR spectrum (Supplementary Material Fig. S4) corroborated the expected structure of *N* ^6^-MF-A to consist of the ribonucleoside with a single 2-methylfuran moiety attached to the exocyclic nitrogen of the adenine, a structural scaffold that has been observed previously for many 2′-deoxyadenosine adducts of SULT-activated hydrocarbons (Herrmann et al. 2012; Monien et al. 2008, 2011, 2012). For the quantification of *N* ^6^-MF-A by UPLC-MS/MS MRM in incubation mixtures containing SULTs and FFA, we synthesized the isotope-labeled reference compound [^15^N_5_]*N* ^6^-MF-A. Figure 3 shows the MRM chromatograms resulting from the neutral loss of the ribose of *N* ^6^-MF-A (*m/z* = 348.1 → 216.1) and the cleavage of the methylfuran moiety (*m/z* = 348.1 → 81.0). As an additional qualifier signal, the release of the *N* ^6^-methylated adenine (*m/z* = 348.1 → 148.0) was monitored, which further increased the specificity of the mass spectrometric detection (not shown). The limit of detection (signal-to-noise ratio = 4) of the technique was 0.8 fmol *N* ^6^-MF-A per injection.

### Sulfoconjugation of FFA by individual SULT forms from human, mouse and rat

The sulfoconjugation assay was performed under similar conditions as used for HMF, with three different concentrations of FFA (0.1, 1 and 10 mM). Table 3 summarizes the formation rates of *N* ^6^-MF-A, which were used as surrogate parameter for the SULT-specific capacities of FFA turnover. In cytosolic preparations containing human SULTs and 1 mM FFA, *N* ^6^-MF-A was most rapidly formed in the presence of SULT1A1, 1C2 and 1B1. Other human enzymes leading to detectable levels of *N* ^6^-MF-A were SULT1A2, 1A3, 1C3, 1E1 and 2A1. Among all mouse Sult forms tested, FFA sulfoconjugation by Sult1a1 and 1d1 led to relatively high levels of *N* ^6^-MF-A, which was less efficiently formed in incubations containing mSult1b1, 1e1, 2a1 and 2a2. In rats, Sult2a3 was an efficient catalyst of FFA sulfoconjugation, whereas rSult1a1, 1b1, 1c1 and 2a1 showed lower, but measurable activity.

**Table 3:** Rates of *N* ^6^-MF-A formation after FFA sulfoconjugation in cytosolic preparations of *S. typhimurium* TA1538 containing different SULT forms and adenosine as nucleophilic scavenger

Species	SULT	Rate (fmol *N* ^6^-MF-A/mg/min)			
		0.1 mM FFA	1 mM FFA	10 mM FFA	1 mM FFA (corr.)^a^
Human	1A1	73.5 ± 1.1	575 ± 2	931 ± 5	57,500 ± 200
	1A2	<18	34.8 ± 0.5	150 ± 4	2,480 ± 30
	1A3	<18	27.3 ± 0.5	226 ± 3	1,950 ± 30
	1B1	23.3 ± 0.3	211 ± 2	1,070 ± 20	5,010 ± 50
	1C1	<18	<20	<44	
	1C2	51.3 ± 0.8	459 ± 2	2,230 ± 10	45,900 ± 200
	1C3	<18	61.8 ± 0.3	248 ± 3	3,090 ± 10^b^
	1E1	<18	<20	47.0 ± 0.9	
	2A1	<18	37.0 ± 0.6	386 ± 6	2,110 ± 40^b^
	2B1a	<18	<20	<44	
	2B1b	<18	<20	<44	
	4A1	<18	<20	<44	
Mouse	1a1	361 ± 4	2,590 ± 20	5,140 ± 60	61,700 ± 400
	1b1	<18	<20	74.3 ± 1.6	
	1c2	<18	<20	<44	
	1d1	52.5 ± 1.7	468 ± 6	2,610 ± 30	4,260 ± 60
	1e1	<18	49.3 ± 1.2	425 ± 3	
	2a1	<18	<20	154 ± 2	
	2a2	<18	<20	216 ± 3	
	2a3	<18	<20	<44	
	2b1b	<18	<20	<44	
	5a1	<18	<20	<44	
Rat	1a1	<18	31.8 ± 0.8	<44	24,400 ± 600
	1b1	<18	34.3 ± 1.0	314 ± 4	343 ± 10
	1c1	<18	97.8 ± 2.3	492 ± 13	3,910 ± 90
	1c2	<18	<20	<44	
	2a1	<18	24.8 ± 1.4	228 ± 7	
	2a3	48.3 ± 1.3	461 ± 8	3,420 ± 30	
	2a4	<18	<20	<44	
	2b1	<18	<20	<44	

Values are mean ± SE of four measurements from independent incubations. Only *N* ^6^-MF-A levels exceeding the background signal by more than twofold were considered to be above the detection limit

^a^The rate of *N* ^6^-MF-A formation at 1 mM FFA was corrected by the actual concentrations of individual SULT forms in the cytosolic preparations (compare Table 2)

^b^If not listed in Table 2, the SULT concentrations were reported in (Meinl et al. 2006)

We determined the reaction efficiency of chemically synthesized 2-sulfoxymethylfuran with adenosine in the presence of cytosolic protein of native *S. typhimurium* TA1538 under normal incubation conditions. In this experiment, about 1.5 % of the 2-sulfoxymethylfuran was converted into *N* ^6^-MF-A (data not shown). However, the estimation was beset with different inaccuracies and, therefore, was not used to determine FFA sulfoconjugation quantitatively. First, the chemical syntheses of 2-sulfoxymethylfuran provide mixtures of different salts, e.g., sodium sulfate, which contained only 18.3 % 2-sulfoxymethylfuran in a previous study (Glatt et al. 2012) and only 13.2 % 2-sulfoxymethylfuran in the current study. Consequently, incubation mixtures of chemically synthesized 2-sulfoxymethylfuran with adenosine did not exactly match the chemical composition of the sulfoconjugation assay. Furthermore, the adduct formation in mixtures of chemically synthesized 2-sulfoxymethylfuran with adenosine may differ from that in incubations in which 2-sulfoxymethylfuran is formed slowly from FFA and SULTs.

The *N* ^6^-MF-A formation rates of the most important SULT forms observed at 1 mM FFA were corrected by the actual SULT level of the cytosolic preparations (Table 2). SULT1A1 was the most efficacious catalyst of FFA sulfoconjugation in human, mouse and rat, leading to formation of 57,500, 61,700 and 24,400 fmol *N* ^6^-MF-A/mg/min, respectively (Table 3). Among the human enzymes, SULT1C2 was also very efficacious (45,900 fmol *N* ^6^-MF-A/mg/min). In contrast, the FFA turnover by other SULT forms was remarkably less effective. The rate of *N* ^6^-MF-A appearance observed in incubation mixtures containing hSULT1A1 was about 11- to 30-fold greater compared with hSULT1A2, 1A3, 1B1, 1C3 and 2A1. Mouse Sult1a1 catalyzed FFA bioactivation about 14 times more effectively compared with mSult1d1, whereas *N* ^6^-MF-A was not detectable at 1 mM FFA in the presence of mSult1b1. In rats, the formation of *N* ^6^-MF-A was about 6- and 71-fold greater in cytosolic preparations containing rSult1a1 compared with those of rSult1c1 and 1b1, respectively. However, the data indicated that hydroxysteroid rSult2a3, which is predominantly expressed in female liver (Dunn and Klaassen 1998), may be the most effective catalyst of FFA sulfoconjugation in rats.

## Discussion

Considerable species-dependent differences of SULT expression and substrate specificities have been found (Glatt 2002). For example, tamoxifen is a potent carcinogen in rat but not in human liver. A major phase I metabolite, α-hydroxytamoxifen is activated by hepatic rat SULT2A3 producing a DNA-reactive sulfate ester (Glatt et al. 1998). Further, the benzylic alcohol 2-hydroxy-3-methylcholanthrene, a primary metabolite of the carcinogen 3-methylcholanthrene, was efficiently bioactivated by hSULT1A1 expressed in *S. typhimurium*, but not by the orthologues of mouse, rat and dog (Meinl et al. 2013). Parallel species-dependent differences may introduce incalculable uncertainties in human risk assessment of SULT-activated compounds from the results of two-year bioassays. The substituted furans HMF and FFA are rodent carcinogens present at high concentrations in the human diet. The carcinogenicity was proposed to originate from sulfoconjugation yielding reactive sulfate esters (Glatt and Sommer 2006; Surh 1998; Surh et al. 1990) that form specific DNA adducts in mammalian cell culture (Monien et al. 2012) and mice (Monien et al. 2011). It is of utmost importance to know whether humans may be more (or less) susceptible to the carcinogenic effects of HMF and FFA intake than rodents.

Here, we studied the sulfoconjugation of the furan derivatives in vitro taking into account twelve human, ten mouse and eight rat SULT forms (Tables 1, 3). Eleven enzymes were selected for detailed kinetic analyses, based on the results of the initial screening and the expression levels in liver (target of HMF-induced carcinogenicity in the mouse), kidney (target of FFA-induced carcinogenicity in the mouse) and intestine (induction of preneoplasia by HMF in rats and mice). In humans, SULT1A1 has a dominant role in the activation of many benzylic alcohols, a broad substrate tolerance, and is highly expressed in numerous tissues (Glatt et al. 2001; Glatt and Meinl 2005). SULT1C2 was of special interest because HMF and FFA were particularly mutagenic in *S. typhimurium* TA100-hSULT1C2 (Glatt et al. 2012), and hSULT1B1 is present at high levels in colon, rectum and in other compartments of the gastrointestinal tract and in the liver (Teubner et al. 2007; Wang et al. 1998), all of which are potential target organs for HMF-induced carcinogenicity (National Toxicology Program 2008; Svendsen et al. 2009, 524; Zhang et al. 1993, 83). SULT1A3 is an abundant enzyme in many tissues (except liver) with especially high levels in the gastrointestinal tract (Teubner et al. 2007).

Mouse Sults were chosen in consideration of the major target tissues of HMF- and FFA-mediated carcinogenic effects in liver (National Toxicology Program 2008) and in kidneys (National Toxicology Program 1999), respectively. Alnouti and Klaassen reported that mSult1a1 is the predominant hepatic form as judged from mRNA levels. Sult1d1 is highly expressed in kidney and at moderate levels throughout the gastrointestinal tract of mice, which is also rich in mSult1b1 (Alnouti and Klaassen 2006). Further, HMF and FFA produced strong mutagenic effects in mSult1a1- and mSult1d1-expressing TA100 strains (Glatt et al. 2012). Rat Sults were selected as follows. In male rats, Sult1a1 and 1c1 are expressed at substantial levels in the liver but also in the kidneys (Dunn and Klaassen 1998; Honma et al. 2001). Rat Sult1b1 is expressed in liver and kidneys of males and females but also in the intestine, a reported target organ for the neoplastic effect of HMF (Zhang et al. 1992).

The catalytic efficiencies of HMF turnover by the selected SULT forms were calculated from Michaelis–Menten constants *K*_M_ and turnover numbers *k*_cat_ (Table 2). Human SULT1A1 and the orthologous enzymes in mice and rats were the predominant catalysts of HMF sulfoconjugation. The data showed that among the other SULT forms only mSult1d1 was similarly effective, but required high substrate concentrations (*K*_M_ = 31.9 ± 5.7 mM). The formation rates of *N* ^6^-MF-A, used as a surrogate marker of FFA sulfoconjugation (Table 3), largely paralleled the ranking of HMF sulfoconjugation capacity. There were two notable exceptions. First, *N* ^6^-MF-A was formed almost equally well by hSULT1C2 and hSULT1A1 in the presence of 1 mM FFA, whereas HMF sulfoconjugation was catalyzed about 19 times more effective by hSULT1A1 compared with hSULT1C2. We did not further investigate this difference, because hSULT1C2 may be of minor importance for the metabolism due to its low expression. The mRNA was primarily found in fetal tissues (Sakakibara et al. 1998), while the hSULT1C2 protein was hitherto detected only in Caco-2 cells (Meinl et al. 2008). The second deviation between HMF and FFA sulfoconjugation was observed for mSul1d1. The catalytic efficiency of mSult1d1 regarding HMF sulfoconjugation was three times less than that observed for mSult1a1, whereas the *N* ^6^-MF-A formation rate in the presence of mSul1d1 at 1 mM FFA was about 14-fold lower compared with that observed for mSult1a1. Apart from these differences, the corrected rates of *N* ^6^-MF-A formation indicated that, in agreement to HMF sulfoconjugation, hSULT1A1 and its rodent orthologues were the most effective catalysts of FFA turnover.

The relative importance of SULT1A1 for the turnover of many other substrates has been reported. For example, a survey of eight human SULTs identified SULT1A1 as the predominant enzyme for the sulfoconjugation of various endogenous compounds, e.g., epinephrine, 2-hydroxyestradiol, T2 (3,5-diiodo-l-thyronine) and cholesterol, as well as xenobiotica, e.g., minoxidil, 4-methylphenol and α-zearalenol (Allali-Hassani et al. 2007). It is of note that the *K*_M_ values of the SULT1A1 forms for the turnover of HMF were high compared with those of other substrates. Honma reported *K*_M_ values of 3.0, 18 and 130 µM for the sulfoconjugation of *p*-nitrophenol, 6-hydroxymelatonin and dopamine by hSULT1A1, respectively (Honma et al. 2001). Similar to HMF, a *K*_M_ of 2.4 mM was determined for the turnover of paracetamol by hSULT1A1 (Adjei et al. 2008). The high *K*_M_ values of all SULT1A1 forms observed in the current study indicate a relatively weak binding affinity of HMF. This is a possible reason why only 500 ppm of 100 mg HMF/kg body weight administered to mice were converted to SMF (Monien et al. 2009). To our knowledge, *K*_M_ values for the conversion of HMF by alcohol and aldehyde dehydrogenases have not been reported.

Due to the lability of the reactive sulfate esters, the bioactivation of benzylic alcohols by individual SULT forms was frequently assessed by the mutagenic effect of the substrates in SULT-expressing *S. typhimurium* TA100 or TA1538 strains (Glatt and Meinl 2004). It was found that hSULT1A1 was most important for the sulfoconjugation of various promutagens, e.g., (±)-1′-hydroxymethyleugenol (Herrmann et al. 2012), nitrofen (Glatt and Meinl 2004), 1-hydroxymethylpyrene (Meinl et al. 2002) and 2-hydroxy-3-methylcholanthrene (Meinl et al. 2013), whereas other SULT forms of human origin or from other species contributed little or nothing at all to the mutagenic activity of these compounds. The results presented in this study were in agreement with a previous report about the mutagenicity of HMF and FFA in *S. typhimurium* TA100 strains expressing different SULT forms. Mutagenic effects of HMF, albeit small, were detectable in those strains that expressed hSULT1A1, 1A3 and 1C2 as well as mSult1a1 and 1d1. The highest mutagenicity of FFA was observed in bacteria expressing hSULT1A1, 1C2 and mSult1a1 (Glatt et al. 2012).

Besides the species-dependent efficiency of SULT1A1-mediated bioactivation, also the expression levels of the enzyme may influence the carcinogenic risk of HMF and FFA in each of the organisms. Despite the principal role of SULT1A1 in the metabolism of many endogenous and exogenous compounds, the knowledge about its expression in different organisms is limited. In humans, SULT1A1 is found in many tissues at high levels, e.g., in liver, lung, brain, throughout the gastrointestinal tract and in kidneys (Glatt 2002; Meinl et al. 2006; Riches et al. 2009; Teubner et al. 2007). The SULT1A1 levels found in human liver (420–4,900 ng/mg cytosolic protein, *n* = 28) (Riches et al. 2009) and the ileum (990–1,600 ng/mg cytosolic protein, *n* = 4) (Teubner et al. 2007) were considerably higher compared with those in kidneys (30–270 ng/mg cytosolic protein, *n* = 10) (Meinl et al. 2006; Riches et al. 2009). Little data of Sult1a1 expression on the protein level are available for mice and rats. Honma et al. reported hepatic Sult1a1 amounts of 2,300 ± 100 ng/mg cytosolic protein (*n* = 3) and 4,400 ± 200 ng/mg cytosolic protein (*n* = 3) in male and female mice, respectively, and 9,100 ± 200 ng/mg cytosolic protein (*n* = 3) and 6,300 ± 500 ng/mg cytosolic protein (*n* = 3) in liver of male and female rats, respectively. Judged from concentrations of mRNA, the highest amounts of mSult1a1 were expressed in liver, lung and large intestine, whereas the Sult1a1 expression in the small intestine of mice was negligible as judged from the mRNA levels (Alnouti and Klaassen 2006). In rats, mRNA of Sult1a1 is expressed in many tissues, albeit at levels that are minute in comparison with the liver (Dunn and Klaassen 1998). Taken together, the current knowledge about SULT1A1 expression in different species does not allow assuming a species-dependent carcinogenic risk of SULT1A1-activated compounds. However, HMF and FFA were more effectively bioactivated by mSult1a1 than by rSult1a1. This may explain why carcinogenic effects of HMF and FFA were observed in mice and not in rats (National Toxicology Program 1999, 2008). Our data further indicate that hSULT1A1 conjugates HMF and FFA as efficient as mSult1a1. However, looking only at SULTs would present a simplification. The concentration of the reactive sulfate esters in particular tissues and species may also be influenced by various other parameters, such as detoxification of the precursors and the sulfate esters, tissue-specific directed export and import of the sulfate esters, transport via the blood stream and excretion.

In summary, it becomes increasingly clear that HMF and FFA are sulfoconjugated not only in mice (Monien et al. 2009, 2011) but also in the human body. The characterization of HMF and FFA sulfoconjugation capacity of individual human, mouse and rat SULT forms showed that hSULT1A1 and the orthologous enzymes in mice and rats catalyzed the bioactivation of the substituted furans most efficiently compared with all other SULTs. Human SULT1A1 was about as effective as Sult1a1 of mice, the principal species in which carcinogenic effects of HMF (liver) and FFA (kidney) were observed. This raises concern because hSULT1A1 is ubiquitously expressed at high levels in several tissues, e.g., liver, lung, gastrointestinal tract and kidney (Glatt 2002; Meinl et al. 2006; Teubner et al. 2007).

## Electronic supplementary material

Below is the link to the electronic supplementary material.

Supplementary material 1 (PDF 203 kb)
