# Supplementary material for: Bioactivation of food genotoxicants 5-hydroxymethylfurfural and furfuryl alcohol by sulfotransferases from human, mouse and rat: a comparative study
Source: Arch Toxicol. 2014 Nov 5;90(1):137–48. doi: 10.1007/s00204-014-1392-6 (PMC4710668; doi:10.1007/s00204-014-1392-6)
Supplement: Supplementary file 1 — Supplementary material 1 (PDF 203 kb) [file 204_2014_1392_MOESM1_ESM.pdf]

Supplementary Material for Archives of Toxicology

Bioactivation of Food Genotoxicants 5-Hydroxymethylfurfural and Furfuryl Alcohol by Sulfotransferases from Human, Mouse and Rat: A Comparative Study

Benjamin Sachse, Walter Meinl, Yasmin Sommer Hansruedi Glatt,

Albrecht Seidel, Bernhard H. Monien[1]

1) corresponding author:

Research Group *Genotoxic Food Contaminants*, German Institute of Human Nutrition (DIfE) Potsdam-Rehbrücke, Arthur-Scheunert-Allee 114-116, 14558 Nuthetal, Germany

phone: +49-33200-882417

fax: +49-33200-882426

email: monien@dife.de

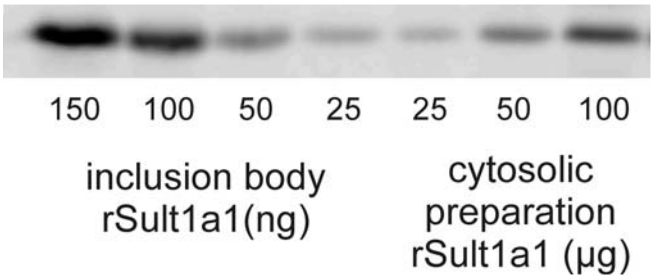

Fig. S1 The SULT content in cytosolic preparations of *S. typhimurium* TA1538 strains was determined using immunoblots with inclusion bodies containing individual SULT forms as standards.

SULT standards

SULT protein standards were prepared from overexpression of individual SULT forms in *Escherichia coli* BL21 DE3 (Novagen, Madison, USA) as described previously (Meinl et al. 2006). Briefly, cDNA of various SULT forms (hSULT1A1, 1A2, 1C2, 1B1, mSult1a1, 1b1 and rSult1a1) were inserted into a high-expression pET28b(1) vector (Novagen), which was transfected into *Escherichia coli* BL21 DE3. The bacteria were grown in the presence of the inducer isopropylthio-β-D-1-galactopyranoside (IPTG). The recombinant strains produced inclusion bodies that appeared to consist exclusively of the corresponding SULT protein, as determined by electrophoresis on sodium dodecylsulphate-polyacrylamide gels and staining with Coomassie blue.

Immunodetection of SULTs

Cytosolic preparations of SULT-expressing *S. typhimurium* TA1538 were separated electrophoretically on an 11 % sodium dodecylsulfate-polyacrylamide gel. Proteins were transferred to a Hybond ECL membrane (GE Healthcare, Munich, Germany) and probed with polyclonal antibodies raised in sheep (against hSULT1A1, hSULT1B1) or rabbit (against hSULT1C2, mSult1a1) at a dilution of 1:10 000. Goat anti-sheep or goat anti-rabbit IgG–peroxidase conjugate (Sigma-Aldrich), at a dilution of 1:2000 -

1:5000, was used as the secondary antibody. The immunoreactive bands were visualized and densitometrically analyzed using an enhanced-chemoluminescence system together with the ImageQuant LAS4000 (GE Healthcare). Inclusion bodies of different SULT forms were included as standards for estimating the SULT concentration in the cytosolic preparations. Fig. S1 in the Supplementary Material depicts a representative immunoblot of different amounts of rSult1a1standard and a cytosolic preparation of *S. typhimurium* expressing rSult1a1. No purified standards were available for mSult1d1, rSult1b1 and rSult1c1, the concentrations of which were estimated from Coomassie gels. After electrophoresis of cytosolic preparations on polyacrylamide gels under denaturing conditions and Coomassie blue staining, an additional protein band was detected in each recombinant strain compared to the control strain. This band, in combination with inclusion bodies of rSult1a1 as standards, was used to estimate the SULT content in cytosolic preparations of *S. typhymurium* TA1538 expressing mSult1d1, rSult1b1 and rSult1c1. Bands were visualized and densitometrically analyzed using the ImageQuant LAS4000 (GE Healthcare).

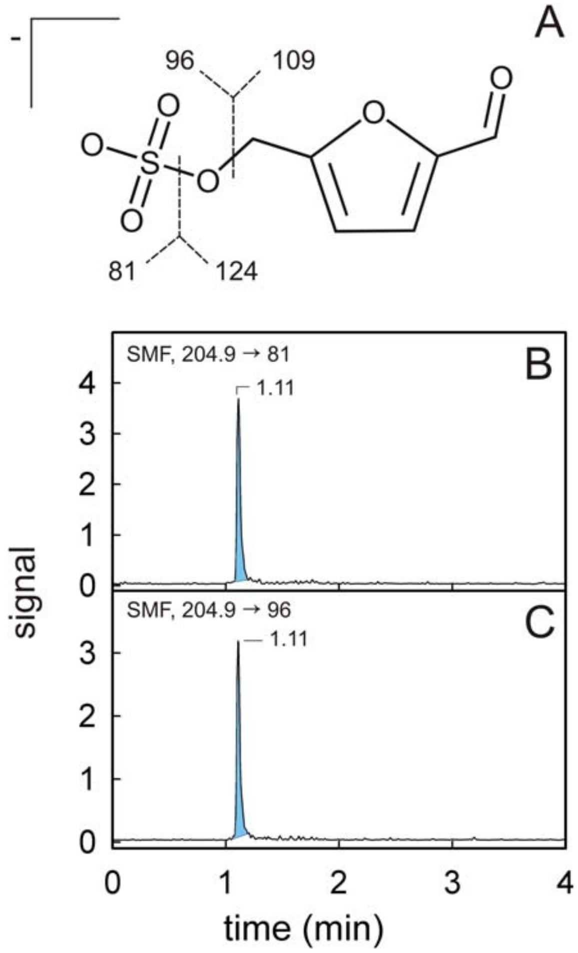

Fig. S2 Analysis of SMF by UPLC-MS/MS multiple reaction monitoring. Collision-induced fragmentation of SMF yielded two principal ions with *m/z* = 96 (sulfate ion radical, $SO_4^{\cdot -}$) and *m/z* = 81 (sulfonate ion, HSO_3^-) (A). The transitions 204.9 → 81 and 204.9 → 96 were used for specific detection and quantification of SMF (B & C). The resulting peaks at 1.11 min were absent in incubation samples lacking HMF.

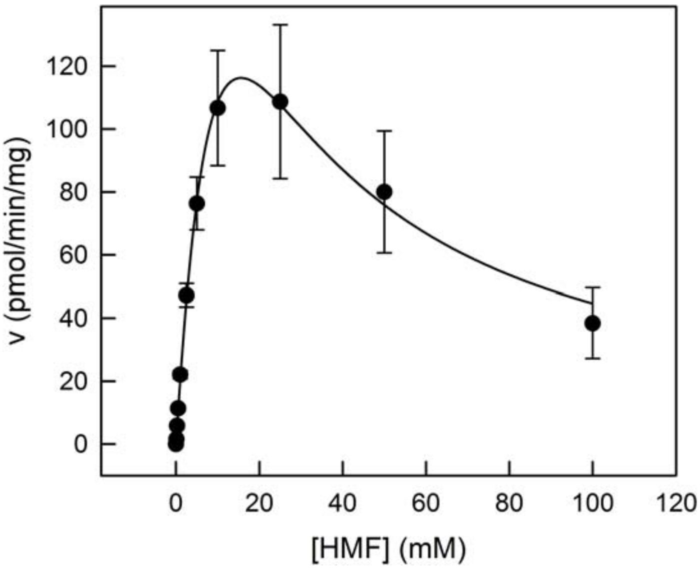

Fig. S3 HMF sulfo conjugation by rat Sult1c1. Rates at single HMF concentrations are means ± SE of four independent measurements. Fitting of the data with a Michaelis-Menten equation considering a competitive inhibitory effect at high substrate concentrations, $V = V_{MAX} /(1 + K_M /[HMF] + [HMF]/ K_I)$, yielded values of K_M = 18.4 ± 4.7 mM, K_I = 13.2 ± 3.4 mM and apparent V_{MAX} = 391 ± 76 pmol/mg/min.

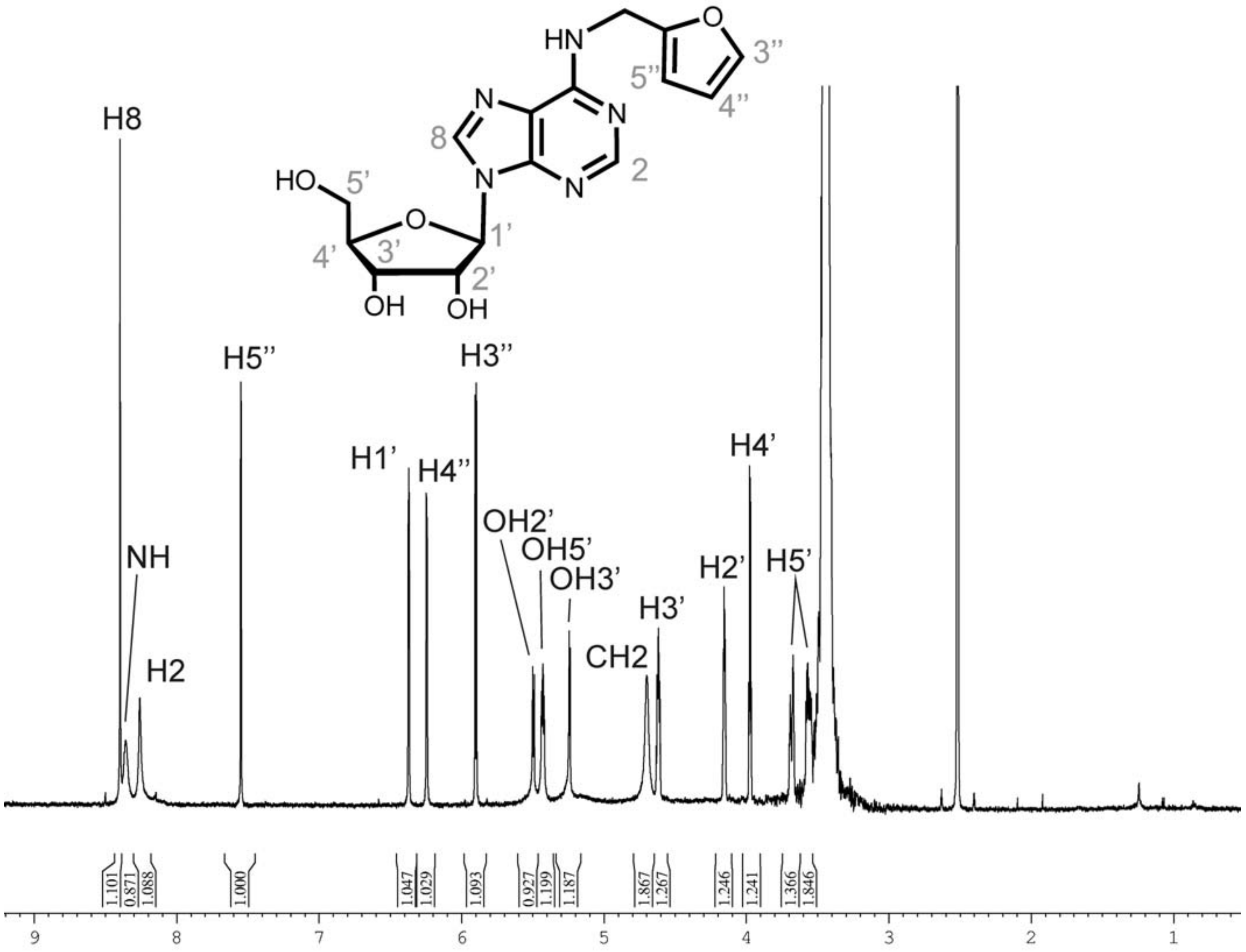

Fig. S4 ^{1}H-NMR spectrum of N^2-MF-A prepared via a nucleophilic substitution from adenosine and 2-sulfoxymethylfuran, and purified by preparative HPLC as described in the method section.

Table S1. Summary of SULT forms used in the current study together with the publications in which the transgenic *S. typhimurium* TA1538 strains were described[a]

hSULT	ref	mSult	ref	rSult	ref
1A1	(Glatt et al. 1998; Meinl et al. 2002)	1a1	(Glatt and Meinl 2004)	1a1	(Glatt et al. 1998)
1A2	(Meinl et al. 2002)	1b1	(Meinl et al. 2013)	1b1	(Meinl et al. 2013)
1A3	(Glatt et al. 1998; Meinl et al. 2002)	1c2	(Bendadani et al. 2014)	1c1	(Glatt and Meinl 2004)
1B1	(Meinl et al. 2002)	1d1	(Bendadani et al. 2014)	1c2	AJ238391
1C1	(Meinl et al. 2002)	1e1	(Bendadani et al. 2014)	2a1[b] (rST-20)	M31363
1C2	(Meinl et al. 2002)	2a1	(Glatt and Meinl 2004)	2a3 (rST-41)	(Glatt et al. 1998)
1C3	(Meinl et al. 2008)	2a2	(Bendadani et al. 2014)	2a4 (rST-60)	D14989
1E1	(Hagen et al. 1998; Meinl et al. 2002)	2a3	(Bendadani et al. 2014)	2b1	AY827148
2A1	(Hagen et al. 1998; Meinl et al. 2002)	2b1b	NM_017465		
2B1a	(Meinl et al. 2002)	5a1	(Bendadani et al. 2014)		
2B1b	(Meinl et al. 2002)				
4A1	(Meinl et al. 2002)				

[a] Details about the preparation of the *S. typhimurium* TA1538 strains and the isolation of SULT-containing cytosols were published by Glatt and Meinl (Glatt et al. 2005) and for some SULT forms as listed separately. The sequence of the coding region of the yet unpublished bacterial strains is identical with the GenBank accession number given.

[b] nomenclature according to Blanchard *et al.* (Blanchard et al. 2004)

Table S2. Comparison of peak areas from UPLC-MS/MS analyses of SMF samples prepared in water or in bacterial cytosol from *S. typhimurium* TA1538 and diluted with 2-propanol

SMF concentration (nM)	matrix [a,b]	average peak area	deviation (%)
10	water	512 ± 16	
10	cytosol	534 ± 8	+ 4.2
25	water	1409 ± 51	
25	cytosol	1313 ± 62	- 6.8
50	water	2749 ± 293	
50	cytosol	2627 ± 86	- 4.4
100	water	5763 ± 940	
100	cytosol	5779 ± 876	+ 0.3
250	water	12231 ± 1171	
250	cytosol	11479 ± 1047	- 6.2

a SMF dissolved in 100 µl water was diluted with 300 µl 2-propanol and subjected to mass spectrometric analysis. Values are means ± SD of six measurements.

b SMF dissolved in 100 µl portions of the incubation medium containing 200 µg bacterial cytosolic protein from unmodified *S. typhimurium* TA1538 was diluted with 300 µl 2-propanol and subjected to mass spectrometric analysis. Values are means ± SD of six measurements.

References

Bendadani C, Meinl W, Monien BH, et al. (2014) Determination of sulfotransferase forms involved in the metabolic activation of the genotoxicant 1-hydroxymethylpyrene using bacterially expressed enzymes and genetically modified mouse models. Chem Res Toxicol 27(6):1060-1069

Blanchard RL, Freimuth RR, Buck J, Weinshilboum RM, Coughtrie MW (2004) A proposed nomenclature system for the cytosolic sulfotransferase (SULT) superfamily. Pharmacogenetics 14(3):199-211

Glatt H, Bartsch I, Christoph S, et al. (1998) Sulfotransferase-mediated activation of mutagens studied using heterologous expression systems. Chem Biol Interact 109(1-3):195-219

Glatt HR, Meinl W (2004) Use of genetically manipulated *Salmonella typhimurium* strains to evaluate the role of sulfotransferases and acetyltransferases in nitrofen mutagenicity. Carcinogenesis 25(5):779-786

Glatt HR, Meinl W (2005) Sulfotransferases and acetyltransferases in mutagenicity testing: technical aspects. Meth Enzymol 400:230-249

Hagen M, Pabel U, Landsiedel R, Bartsch I, Falany CN, Glatt H (1998) Expression of human estrogen sulfotransferase in Salmonella typhimurium: differences between hHST and hEST in the enantioselective activation of 1-hydroxyethylpyrene to a mutagen. Chem Biol Interact 109(1-3):249-253

Meinl W, Donath C, Schneider H, Sommer Y, Glatt H (2008) SULT1C3, an orphan sequence of the human genome, encodes an enzyme activating various promutagens. Food Chem Toxicol 46(4):1249-1256

Meinl W, Meerman JH, Glatt HR (2002) Differential activation of promutagens by alloenzymes of human sulfotransferase 1A2 expressed in *Salmonella typhimurium*. Pharmacogenetics 12(9):677-689

Meinl W, Pabel U, Osterloh-Quiroz M, Hengstler JG, Glatt HR (2006) Human sulfotransferases are involved in the activation of aristolochic acids and are expressed in renal target tissue. Int J Cancer 118:1090-1097

Meinl W, Tsoi C, Swedmark S, Tibbs ZE, Falany CN, Glatt H (2013) Highly selective bioactivation of 1- and 2-hydroxy-3-methylcholanthrene to mutagens by individual human and other mammalian sulphotransferases expressed in *Salmonella typhimurium*. Mutagenesis 28(5):609-619
